# Admissible Consensus for Descriptor Multi-Agent Systems with Exogenous Disturbances

**DOI:** 10.3390/e20040276

**Published:** 2018-04-12

**Authors:** Xuxi Zhang, Siqi Wang

**Affiliations:** College of Science, Harbin Engineering University, Harbin 150001, China

**Keywords:** descriptor multi-agent systems, exogenous disturbances, disturbance observer

## Abstract

In this paper, we study the admissible consensus for descriptor multi-agent systems (MASs) with exogenous disturbances that are generated by some linear systems. The topology among agents is represented by a directed graph. For solving the admissible consensus problem, the exogenous disturbance observer and distributed control protocol are proposed. With the help of the graph theory and the generalized Riccati equation, some conditions for admissible consensus of descriptor MASs with exogenous disturbances are obtained. Finally, we provide a numerical simulation to effectively illustrate the results we have reached before.

## 1. Introduction

The descriptor multi-agent systems (MASs) is a combination of the descriptor system and the MASs. The descriptor system was first mentioned in [1], and is also referred to as generalized system, singular system, or implicit system. Since its introduction, it has been widely used [2,3]. For MASs, its definition is given in [4], and its application has also become more and more widespread in real life [5], industry [6] and technology [7,8] with the rapid development of science and technology. Moreover, the three-link manipulators has been taken as an example of the descriptor MASs in [9].

As is well known, the consensus problem is one of the hot topics of coordinated control theory [10,11,12,13,14]. The main task of the consensus problem is to design distributed protocol according to the information obtained from neighbors, so that the states of a group of agents can reach an agreement. Until now, there exist many studies of the consensus problem of MASs. For example, the first-order system consensus problem [15,16], second-order system consensus problem [17], general system consensus problem [18,19,20]. For instance, in [16], the average consensus problem was explored for the first order multi-agent systems with uncertain topologies as well as multiple time-varying delays; in [18], the consensus problem was addressed for general linear multi-agent systems with a time-invariant communication topology. In addition, the admissible consensus problem of the descriptor MASs was further studied in [21]. The conditions of consensus-ability with respect to a set of admissible consensus protocols were investigated in [9]. It is worth mentioning that the observer-based design approach of [19] that can solve the consensus problem of general system was generalized to resolve the admissible consensus problem in [22]. In [23], a distributed observer is used to solve the admissible consensus problem of descriptor MAS, and a new framework is proposed.

However, in the real life, the exogenous disturbances exist and cannot be neglected, for example, the wind, and the electromagnetic interference. Therefore, in this paper, we study the admissible consensus problem of the descriptor MASs with exogenous disturbances. Many researchers have studied the consensus problem of MASs with exogenous disturbance [24,25,26,27,28,29]. For example, in [28], group consensus problems of first order dynamic agents in the presence of random noises and communication delays were investigated, while $L^{1}$ group consensus problem of discrete-time multi-agent systems with external stochastic inputs was studied in [29]. In addition, [30] also studied the consensus problem of linear MASs with exogenous disturbances generated from heterogeneous exosystems. However, there is little research on the admissible consensus problem for descriptor MASs with exogenous disturbances. For example, [31] only studied cooperative output regulation of singular heterogeneous MASs. This situation also appears in other literature, and there are few relevant results on this issue. Therefore, in this paper, we further study the admissible consensus problem of descriptor MASs with exogenous disturbances. The main contributions of this paper are (1) Consider a descriptor MASs with exogenous disturbances, the consensus problem of general linear system is extended to the descriptor MASs; (2) The disturbance observer for descriptor MASs is designed by state feedback; (3) The disturbance observer is used for disturbance attenuation.

The rest of the paper is organized as follows: In Section 2, we introduce some basic concepts and related theorems to describe descriptor MASs. Then, the formulation of the considered problem is introduced. In Section 3, the disturbance observer is proposed, the corresponding distributed control protocol is designed and sufficient conditions that ensure the states of a group of agents can reach an agreement are obtained. Section 4 gives a numerical example. Finally, conclusions are given in Section 5.

## 2. Preliminaries and Problem Formulation

### 2.1. Preliminaries

In this subsection, some notations and preliminaries involved in this paper are introduced. $I_{n}$ denotes the n×n identity matrix. For a matrix *A* (or a vector *x*), $A^{T}$ (or $x^{T}$) represents the transpose of *A* (or *x*). Let σ(A) be the set of all eigenvalues of the square matrix *A*, and let σ(E,A) be {λ|λ∈C,det(λE-A)=0}. $C^{-}$ represents the open left-half complex plane. Let ⊗ denotes the Kronecker product of matrix $A\in R^{m\times n}$ and $B\in R^{p\times q}$, which is defined as

$$A⊗B=\left[\begin{array}{ccc} a_{11}B & \cdots & a_{1n}B\\ \vdots & \ddots & \vdots \\ a_{m1}B & \cdots & a_{mn}B \end{array}\right]$$

and satisfies the following properties

(A⊗B)(C⊗D)=(AC)⊗(BD)

A⊗B+A⊗C=A⊗(B+C)

In this paper, the information interaction topology is modeled by a weighted digraph G = {V,ε,A}, where $V=\{v_{1},v_{2},\cdots ,v_{n}\}$ is the set of vertices, ε⊂V×V is the set of edges, $A=\left[a_{ij}\right]\in R^{n\times n}$ is a weighted adjacency matrix. An edge of G that is from node $v_{i}$ to node $v_{j}$ is denoted by $\left(v_{i},v_{j}\right)$, which represents node $v_{j}$ can get information from node $v_{i}$, but not necessarily vice versa. The set of neighbors of node $v_{i}$ is $N_{i}=\left\{v_{j}|\left(v_{j},v_{i}\right)\in \varepsilon \right\}$. $a_{ij}$ denotes the weight of the edge $\left(v_{j},v_{i}\right)$ and $a_{ii}=0,a_{ij}>0\Leftrightarrow \left(v_{j},v_{i}\right)\in \varepsilon$. The degree matrix $D=diag\left\{d_{1},\cdots ,d_{n}\right\}\in R^{n\times n}$ of digraph G is a diagonal matrix with $d_{i}=\sum_{j=1}^{n}a_{ij}$. Then, the Laplacian matrix of G is defined as L=D-A, which has at least one zero eigenvalue with $1={\left[1,1,\cdots ,1\right]}^{T}$ as its corresponding right eigenvector. In addition, *L* has exactly one zero eigenvalue if and only if the directed graph G contains a directed spanning tree.

**Definition** **1.** *[21] Let E, $A\in R^{n\times n}$. (i) The pair (E,A) is said to be regular if det(sE-A) is not identically zero for some s∈C; (ii) The pair (E,A) is said to be impulse free if (E,A) is regular and deg(det(sE-A))=rankE for ∀s∈C; (iii) The pair (E,A) is said to be stable if $\sigma \left(E,A\right)\subseteq C^{-}$; (iv) The pair (E,A) is said to be admissible if (E,A) is impulse free and stable.*

**Definition 2.**  *(E,A,B) is stable, if there is a matrix K that satisfies $\sigma \left(E,A+BK\right)\subset C^{-}$.*

**Definition 3.**  *(E,A,C) is detectable, if there is a matrix L that satisfies $\sigma \left(E,A+LC\right)\subset C^{-}$.*

### 2.2. Problem Formulation

In this subsection, we will establish the model of the descriptor MASs with exogenous disturbances and propose the corresponding consensus problem for this system. In addition, the corresponding model of the exogenous disturbances system is also given.

Consider a descriptor MASs composing of *n* identical agents. The dynamic of agent *i* is modeled by the following descriptor linear system

(1) $$\begin{array}{c} E{\dot{x}}_{i}\left(t\right)=Ax_{i}\left(t\right)+B\left(u_{i}\left(t\right)+\omega_{i}\left(t\right)\right),\\ y_{i}\left(t\right)=Cx_{i}\left(t\right)+D\omega_{i}\left(t\right),i=1,2,\cdots ,n, \end{array}$$

where $x_{i}\left(t\right)\in R^{m}$ is the state variable, $y_{i}\left(t\right)\in R^{q}$ is the measured output, $u_{i}\left(t\right)\in R^{p}$ is the control input, $\omega_{i}\left(t\right)\in R^{p}$ is exogenous disturbance. *E*, *A*, *B*, *C*, *D* are real constant matrices with appropriate dimensions, and rankE=r<m.

Since *E* is a singular matrix, the initial state cannot be arbitrarily chosen, which needs to satisfy some algebraic conditions.

The descriptor MASs (1) is said to achieve consensus, if the states of all agents satisfy

(2) $$\lim_{t\to \infty }\left(x_{j}-x_{i}\right)=0,\;i,j=1,2,\cdots ,n,$$

for any initial states $x_{i}\left(0\right)$, i=0,1,⋯,n.

If the closed-loop system is admissible and achieves consensus, we say the protocol $u_{i}\left(i=1,2,\cdots ,n\right)$ can solve the admissible consensus problem.

In this paper, we suppose that the disturbance $\omega_{i}\left(t\right)$ is generated by the following linear exogenous system

(3) $${\dot{\xi }}_{i}\left(t\right)=G\xi_{i}\left(t\right),$$

(4) $$\omega_{i}\left(t\right)=F\xi_{i}\left(t\right),$$

where $\xi_{i}\left(t\right)\in R^{l}$ is the state of the exogenous system, $G\in R^{l\times l}$ and $F\in R^{p\times l}$ are the matrices of the disturbance system.

**Lemma** **1.** *[21] Assume that (E,A) is impulse free and (E,A,B) is stable. Then, the generalized Riccati equation*

(5) $$A^{T}P+P^{T}A-P^{T}BB^{T}P+I_{n}=0$$

$$E^{T}P=P^{T}E\geq 0$$

*has at least one admissible solution P, that is, $\left(E,A-BB^{T}P\right)$ is admissible. Furthermore, the admissible solution P is unique in the sense of $E^{T}P$.*

**Lemma** **2.** *[9] Assume (E,A,C) is detectable, and the matrix P is the admissible solution of the following Riccati equation*

(6) $$A^{T}P+P^{T}A-P^{T}BB^{T}P+CC^{T}=0$$

$$E^{T}P=P^{T}E\geq 0.$$

*Then, $\sigma \left(E,A-\left(a+bi\right)BB^{T}P\right)\subset C^{-}$, when $a\geq \frac{1}{2}$, b∈R, where i is imaginary units.*

## 3. Main Results

In this section, we will design a disturbance observer to solve the problem caused by exogenous disturbances. Moreover, in order to resolve admissible consensus problem, we propose the distributed consensus protocol.

The disturbance observer is designed as follows

(7) $$\begin{array}{c} {\dot{\eta }}_{i}=\left(G+HBF\right)\left(\eta_{i}-HEx_{i}\right)+H\left(Ax_{i}+Bu_{i}\right),\\ {\hat{\xi }}_{i}=\eta_{i}-HEx_{i},\\ {\hat{\omega }}_{i}=F{\hat{\xi }}_{i}, \end{array}$$

where $\eta_{i}\in R^{l}$ is the internal state variable of the observer, ${\hat{\xi }}_{i}$ and ${\hat{\omega }}_{i}$ are the estimated values of $\xi_{i}$ and $\omega_{i}$, respectively. $H\in R^{l\times m}$ is the gain matrix of the observer.

Let $e_{i}=\xi_{i}-{\hat{\xi }}_{i}$, we can get

$$\begin{array}{cc} {\dot{e}}_{i} & ={\dot{\xi }}_{i}-{\dot{\hat{\xi }}}_{i}\\ & =G\xi_{i}-\left(\dot{\eta_{i}}-HE\dot{x_{i}}\right)\\ & =G\xi_{i}-\left[\left(G+HBF\right)\left(\eta_{i}-HEx_{i}\right)+H\left(Ax_{i}+Bu_{i}\right)-H\left(Ax_{i}+Bu_{i}+BF\xi_{i}\right)\right]\\ & =G\xi_{i}-G\eta_{i}+GHEx_{i}-HBF\eta_{i}+HBFHEx_{i}+HBF\xi_{i}\\ & =G\left(\xi_{i}-\eta_{i}+HEx_{i}\right)+HBF\left(\xi_{i}-\eta_{i}+HEx_{i}\right)\\ & =\left(G+HBF\right)\left(\xi_{i}-{\hat{\xi }}_{i}\right)\\ & =\left(G+HBF\right)e_{i}. \end{array}$$

That is

(8) $${\dot{e}}_{i}=\left(G+HBF\right)e_{i}.$$

Denoting $e={\left(e_{1}^{T},e_{2}^{T},\cdots ,e_{n}^{T}\right)}^{T},$ then, one has

(9) $$\left(I_{n}⊗I_{l}\right)\dot{e}=\left[I_{n}⊗\left(G+HBF\right)\right]e.$$

We can get that the tracking errors $e_{i},i=1,2,\;\ldots ,n$, converge to 0, when the matrix G+HBF is Hurwitz.

In order to resolve admissible consensus problem, we consider the following distributed consensus protocol

(10) $$u_{i}=K\sum_{j\in N_{i}}a_{ij}\left(x_{j}-x_{i}\right)-F{\hat{\xi }}_{i},\;i=1,2,\cdots ,n$$

where *K* is the gain matrix to be designed.

**Theorem** **1.** *For the descriptor MASs (1) whose interaction topology G contains a directed spanning tree, suppose that the pair (E,A) is regular and impulse free, (E,A,B) is stable, and (E,A,C) is detectable. Then, the protocol (10) can solve the consensus problem if*

*(i)*  *The matrix G+HBF is Hurwitz;*
*(ii)*  *$K=\alpha B^{T}P$, where matrix P is the unique admissible solution of (5) and $\alpha \geq \frac{1}{2min_{\lambda_{i}\left(L\right)\neq 0}\left\{Re\left(\lambda_{i}\right)\right\}}$, i = 1,2,⋯,n.*

**Proof.**  By substituting the control protocol (10) into the system (1), one has

(11) $$\begin{array}{cc} E{\dot{x}}_{i} & =Ax_{i}+Bu_{i}+BF\xi_{i}\\ & =Ax_{i}+BK\sum_{j\in N_{i}}a_{ij}\left(x_{j}-x_{i}\right)+BFe_{i}. \end{array}$$

Next, we write formula (11) in the following form

(12) $$\begin{array}{cc} \left[\begin{array}{c} E{\dot{x}}_{1}\\ E{\dot{x}}_{2}\\ \vdots \\ E{\dot{x}}_{n} \end{array}\right] & =\left[\begin{array}{c} Ax_{1}+BK\sum_{j\in N_{1}}a_{1j}\left(x_{j}-x_{1}\right)+BFe_{1}\\ Ax_{2}+BK\sum_{j\in N_{2}}a_{2j}\left(x_{j}-x_{2}\right)+BFe_{2}\\ \vdots \\ Ax_{n}+BK\sum_{j\in N_{n}}a_{nj}\left(x_{j}-x_{n}\right)+BFe_{n} \end{array}\right]\\ & =\left[\begin{array}{c} Ax_{1}\\ Ax_{2}\\ \vdots \\ Ax_{n} \end{array}\right]+\left(\left[\begin{array}{cccc} -\sum_{j\in N_{1}}a_{1j} & a_{12} & \cdots & a_{1n}\\ a_{21} & -\sum_{j\in N_{2}}a_{2j} & \cdots & a_{2n}\\ \vdots & \vdots & \ddots & \vdots \\ a_{n1} & a_{n2} & \cdots & -\sum_{j\in N_{n}}a_{nj} \end{array}\right]⊗BK\right)\left[\begin{array}{c} x_{1}\\ x_{2}\\ \vdots \\ x_{n} \end{array}\right]+\left[\begin{array}{c} BFe_{1}\\ BFe_{2}\\ \vdots \\ BFe_{n} \end{array}\right] \end{array}$$

Denote $x={\left(x_{1}^{T},x_{2}^{T},\cdots ,x_{n}^{T}\right)}^{T}$, $e={\left(e_{1}^{T},e_{2}^{T},\cdots ,e_{n}^{T}\right)}^{T}$. Then, it follows that

(13) $$\left(I_{n}⊗E\right)\dot{x}=\left(I_{n}⊗A-L⊗BK\right)x+\left(I_{n}⊗BF\right)e$$

Then, after manipulations with combining (9) and (13), the closed-loop system can be expressed as

(14) $$\begin{array}{cc} & \left[\begin{array}{cc} I_{n}⊗E & 0\\ 0 & I_{n}⊗I_{l} \end{array}\right]\left[\begin{array}{c} \dot{x}\\ \dot{e} \end{array}\right]\\ = & \left[\begin{array}{cc} I_{n}⊗A-L⊗BK & I_{n}⊗BF\\ 0 & I_{n}⊗\left(G+HBF\right) \end{array}\right]\left[\begin{array}{c} x\\ e \end{array}\right]. \end{array}$$

Since the topology G contains a directed spanning tree, *L* has exactly one zero eigenvalue. Let $r^{T}=\left(r_{1},r_{2},\cdots ,r_{n}\right)$ be the left zero eigenvector of *L* with $r^{T}1=1$. With the help of the Jordan decomposition theory, there exists a transformation matrix *S* with form $S=[1,S_{1}]$ and $S^{-1}=\left[\begin{array}{c} r^{T}\\ Q_{1} \end{array}\right]$ such that $S^{-1}LS=\left[\begin{array}{cc} 0 & 0\\ 0 & \Lambda \end{array}\right]$, where Λ is the Jordan block diagonal matrix. The diagonal entries of the upper triangular matrix Λ are non-zero eigenvalue of *L*.

Furthermore, define the following state transformation:

$$\begin{array}{cc} \bar{x} & =(S^{-1}⊗I_{m})x,\\ \bar{e} & =(S^{-1}⊗I_{l})e. \end{array}$$

Then, (14) can be divided into the following two subsystems

(15) $$\begin{array}{c} E{\dot{\bar{x}}}^{0}=A{\bar{x}}^{0}+BF{\bar{e}}^{0}\\ {\dot{\bar{e}}}^{0}=\left(G+HBF\right){\bar{e}}^{0} \end{array}$$

and

(16) $$\begin{array}{cc} & \left[\begin{array}{cc} I_{n-1}⊗E & 0\\ 0 & I_{n-1}⊗I_{l} \end{array}\right]\left[\begin{array}{c} {\dot{\bar{x}}}^{1}\\ {\dot{\bar{e}}}^{1} \end{array}\right]\\ = & \left[\begin{array}{cc} I_{n-1}⊗A-\Lambda ⊗BK & I_{n-1}⊗BF\\ 0 & I_{n-1}⊗\left(G+HBF\right) \end{array}\right]\left[\begin{array}{c} {\bar{x}}^{1}\\ {\bar{e}}^{1} \end{array}\right], \end{array}$$

where $\bar{x}={\left[{\bar{x}}^{0T},{\bar{x}}^{1T}\right]}^{T}$, $\bar{e}={\left[{\bar{e}}^{0T},{\bar{e}}^{1T}\right]}^{T}$, and ${\bar{x}}^{0T}$ is the first *m* components of $\bar{x}$, ${\bar{e}}^{0T}$ is the first *l* components of $\bar{e}$.

At the same time, we have

(17) $$\begin{array}{c} x\left(t\right)-1⊗{\bar{x}}^{0}\left(t\right)\\ =\left(S⊗I_{m}\right)\bar{x}\left(t\right)-1⊗{\bar{x}}^{0}\left(t\right)\\ =\left[1⊗I_{m}\;S_{1}⊗I_{m}\right]\left[\begin{array}{c} {\bar{x}}^{0}\left(t\right)\\ {\bar{x}}^{1}\left(t\right) \end{array}\right]-1⊗{\bar{x}}^{0}\left(t\right)\\ =\left(S_{1}⊗I_{m}\right){\bar{x}}^{1}\left(t\right). \end{array}$$

Obviously, if the system (16) is admissible, we can get ${\bar{x}}^{1}\left(t\right)\to 0$, as t→∞. Thus, $x\left(t\right)-1⊗{\bar{x}}^{0}\left(t\right)\to 0$, as t→∞, which means that $\lim_{t\to \infty }\left(x_{j}-x_{i}\right)=0,i,j=1,2,\;\ldots ,n$.

Next, we will prove that the descriptor system (16) is admissible. Due to the matrix G+HBF is Hurwitz, the admissibility of the system (16) is equivalent to the admissibility of all pairs $\left(E,A-\lambda_{i}BK\right)$. According to Lemma 1, *P* is the unique admissible solution of (5), therefore, $\left(E,A-BB^{T}P\right)$ is admissible. Due to $A-\lambda_{i}BK=A-\alpha \lambda_{i}BB^{T}P$, where $Re\left(\alpha \lambda_{i}\right)\geq \frac{1}{2},i=\left\{2,3,\;\ldots ,n\right\}$, according to Lemma 2, $\left(E,A-\lambda_{i}BK\right)\left(i=\left\{2,3,\;\ldots ,n\right\}\right)$ is admissible. So far, the proof is finished. ☐

## 4. Simulations

**Figure 1:**
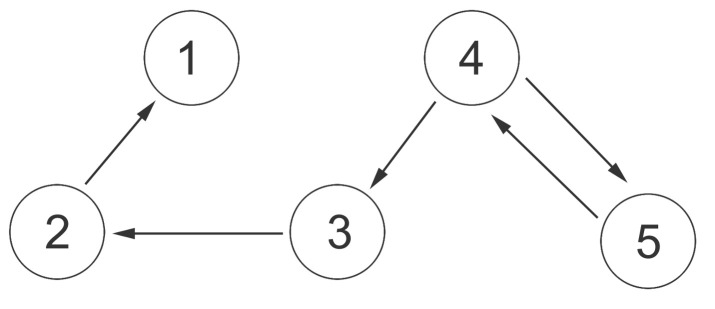
Communication topology.

In this section, a simulation result is presented to illustrate the previous theoretical results. The network includes five agents. The topology can be described in Figure 1. The following matrix is the weighted adjacency matrix A of the topology:

$$A=\left[\begin{array}{ccccc} 0 & 1 & 0 & 0 & 0\\ 0 & 0 & 1 & 0 & 0\\ 0 & 0 & 0 & 1 & 0\\ 0 & 0 & 0 & 0 & 1\\ 0 & 0 & 0 & 2 & 0 \end{array}\right].$$

Choosing matrices

$$\begin{array}{c} E=\left[\begin{array}{ccc} 1 & 0 & 0\\ 0 & 1 & 0\\ 0 & 0 & 0 \end{array}\right],\;A=\left[\begin{array}{ccc} 0.7 & 3.3 & -1.3\\ 3.3 & 4.3 & -3.1\\ -1.3 & -2.2 & 0.8 \end{array}\right],\;B=\left[\begin{array}{c} 1\\ 2\\ -1 \end{array}\right] \end{array}$$

$$\begin{array}{c} G=\left[\begin{array}{ccc} -1 & 0 & 0\\ 0 & -0.6 & 0\\ 0 & 0 & 0 \end{array}\right],\;H=\left[\begin{array}{ccc} 2 & 0 & 2\\ 0 & 1 & 0\\ 0 & 0 & 1 \end{array}\right],\;F=\left[0\;0\;1\right]. \end{array}$$

Solving the Equation (5), we obtain that

$$P=\left[\begin{array}{ccc} 1 & 1 & 0\\ 1 & 2 & 0\\ 0 & 0 & 2 \end{array}\right].$$

Then, we have

α=1

and

$$K=\left[\begin{array}{ccc} 3 & 5 & -2 \end{array}\right].$$

For the simulation, let initial states of the agents be the following:

$$x_{1}=\left[\begin{array}{c} 1\\ 0.5\\ 279/468 \end{array}\right],\;x_{2}=\left[\begin{array}{c} 0.5\\ 2\\ 711/156 \end{array}\right],\;x_{3}=\left[\begin{array}{c} -2\\ 2.5\\ 861/312 \end{array}\right],\;x_{4}=\left[\begin{array}{c} -0.5\\ -1\\ -353/104 \end{array}\right],\;x_{5}=\left[\begin{array}{c} -1\\ 1\\ 328/416 \end{array}\right].$$

**Figure 2:**
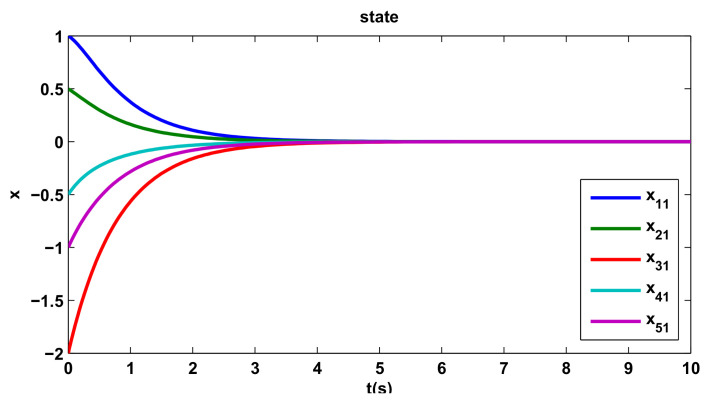
The first component of the states.

**Figure 3:**
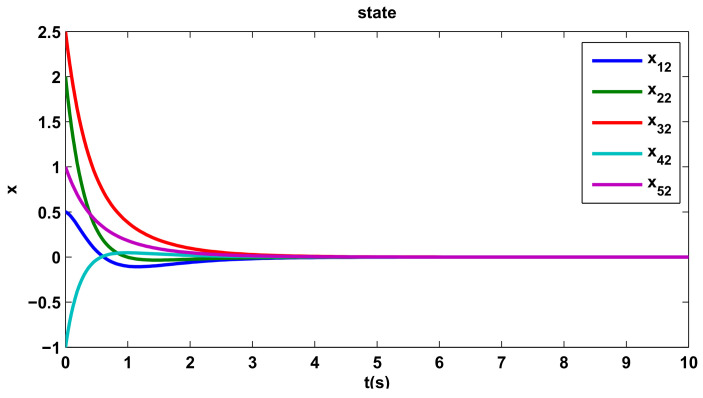
The second component of the states.

**Figure 4:**
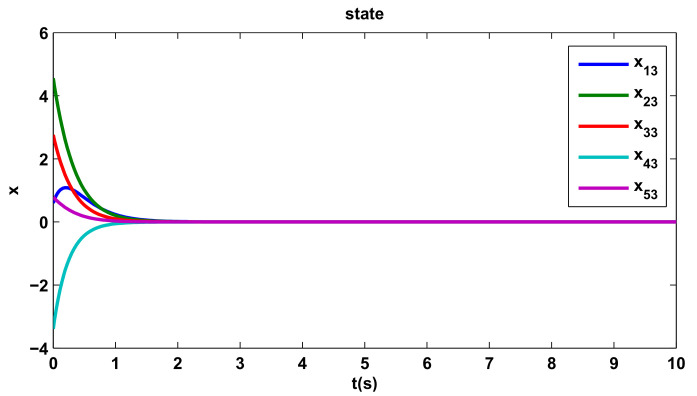
The third component of the states.

Figure 2, Figure 3 and Figure 4 show the states trajectory for all agents, indicating that the states trajectory of agents reach an agreement by the distributed control protocol (10). If we take the $x_{13}$ of Figure 4 as an example, although its initial state and direction of movement are very different from those of other agents, they can eventually reach the consensus agreement by the distributed control protocol (10).

**Figure 5:**
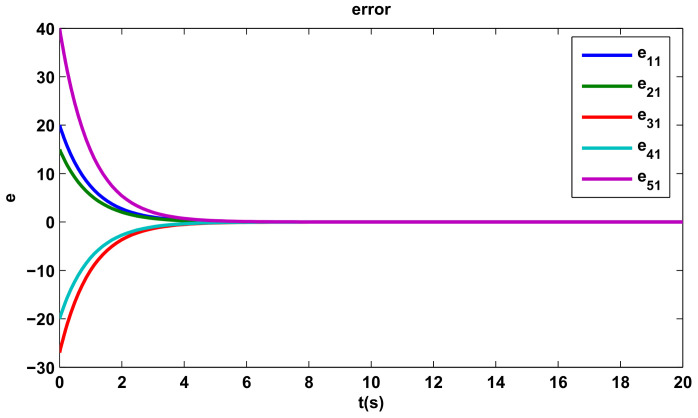
The first component of tracking errors.

**Figure 6:**
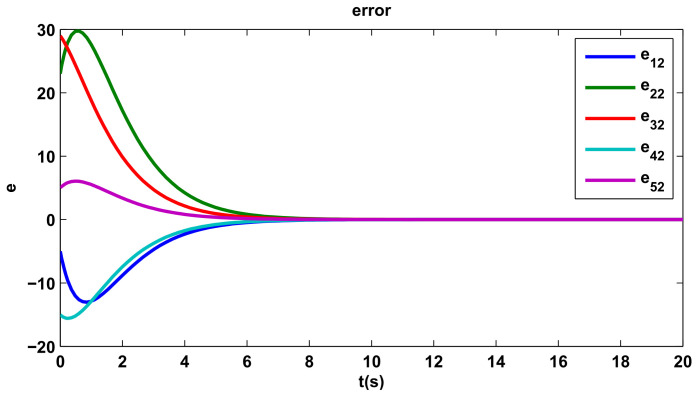
The second component of tracking errors.

**Figure 7:**
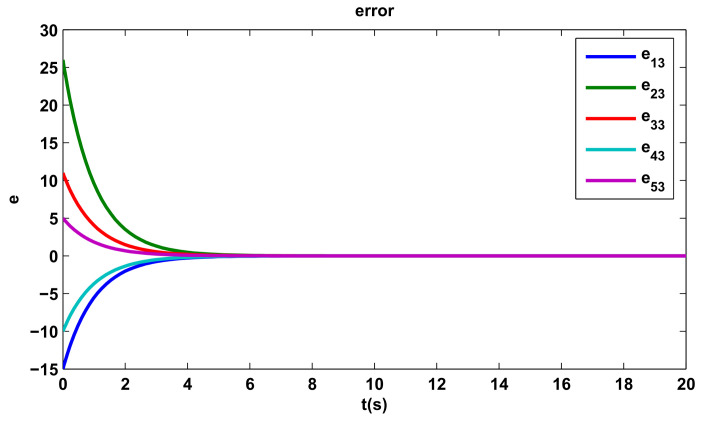
The third component of tracking errors.

The time evolutions of tracking errors are depicted in Figure 5, Figure 6 and Figure 7, we can get that the tracking errors converge to zero asymptotically.

## 5. Conclusions

In this paper, we study the admissible consensus for descriptor MASs with exogenous disturbances. A design method of disturbance observer is proposed, so that the states of all agents reach an agreement. Through the use of graph theory and the generalized Riccati equation, some conditions were obtained for admissible consensus of descriptor MASs with exogenous disturbances. The future work is to consider the admissible consensus of the descriptor MASs with leaders. In addition, since this paper does not consider the time delay, the perspective of considering the time delay will also be an interesting topic.
